# Do guidelines influence the implementation of health programs? — Uganda’s experience

**DOI:** 10.1186/1748-5908-7-98

**Published:** 2012-10-15

**Authors:** Juliet Nabyonga Orem, Juliet Bataringaya Wavamunno, Solome K Bakeera, Bart Criel

**Affiliations:** 1 Health systems and services cluster, WHO Uganda office, P. O. Box 24578, Kampala, Uganda; 2 Health Policy and Planning Department, School of Public Health, Makerere University, P. O. Box 7072, Kampala, Uganda; 3 Public Health Department Institute of Tropical Medicine, Nationalestraat 155, Antwerpen, 2000, Belgium

**Keywords:** Guidelines, Implementation, Health services, Planning, Management, Uganda

## Abstract

**Background:**

A guideline contains processes and procedures intended to guide health service delivery. However, the presence of guidelines may not guarantee their implementation, which may be a result of weaknesses in the development process. This study was undertaken to describe the processes of developing health planning, services management, and clinical guidelines within the health sector in Uganda, with the goal of understanding how these processes facilitate or abate the utility of guidelines.

**Methods:**

Qualitative and quantitative research methods were used to collect and analyze data. Data collection was undertaken at the levels of the central Ministry of Health, the district, and service delivery. Qualitative methods included review of documents, observations, and key informant interviews, as well as quantitative aspects included counting guidelines. Quantitative data were analyzed with Microsoft Excel, and qualitative data were analyzed using deductive content thematic analysis.

**Results:**

There were 137 guidelines in the health sector, with programs related to Millennium Development Goals having the highest number (n = 83). The impetus for guideline development was stated in 78% of cases. Several guidelines duplicated content, and some conflicted with each other. The level of consultation varied, and some guidelines did not consider government-wide policies and circumstances at the service delivery level. Booklets were the main format of presentation, which was not tailored to the service delivery level. There was no framework for systematic dissemination, and target users were defined broadly in most cases. Over 60% of guidelines available at the central level were not available at the service delivery level, but there were good examples in isolated cases. There was no framework for systematic monitoring of use, evaluation, and review of guidelines. Suboptimal performance of the supervision framework that would encourage the use of guidelines, assess their utilization, and provide feedback was noted.

**Conclusions:**

Guideline effectiveness is compromised by the development process. To ensure the production of high-quality guidelines, efforts must be employed at the country and regional levels. The regional level can facilitate pooling resources and expertise in knowledge generation, methodology development, guideline repositories, and capacity building. Countries should establish and enforce systems and guidance on guideline development.

## Background

Although a guideline has been defined in several ways, the free online dictionary defines it as a rule or principle that provides guidance to appropriate behavior [1]. It has also been defined as a document that aims to streamline particular processes according to a set routine [2], and as a document that contains recommendations about health interventions, whether they be clinical, public health-related, or policy interventions [3]. In this article, we define a guideline as a written document containing processes and procedures to guide health service delivery and management that is issued by the Ministry of Health (MoH). Guidelines are developed for various reasons, including: to bridge the gap between evidence and practice; to minimize variations in practice; to improve health outcomes; to improve quality of care; to reduce costs; where the topic is complex; and in cases where valid guidelines are lacking [4-8]. Evidence of inconsistencies between available research and expert recommendations and practice has raised the demand for guidelines to be informed by the best available evidence [8,9].

Research is undertaken to identify solutions to complex health problems and health system challenges, and it must be translated into practical recommendations that are then implemented. Guidelines can be used as a knowledge source, but also as a way to translate evidence into practice [10]. This is even more important in low-income countries, where resources are scarce emphasizing the need for evidence-informed decisions. However, the presence of guidelines may not guarantee their implementation or utility, and some studies have documented the failure of guidelines to influence the implementation of health programs [7,11-13]. Much of the published work that reviews the utility of guidelines stems from a clinical perspective [12,14]. Health services planning and management is a relatively new discipline, and much less work has been carried out on the subject [5]. The increase in the production of guidelines in health services planning and management will likely occur as the discipline matures. For example, the number of guideline documents on the websites of the World Health Organization (WHO) and other agencies has increased significantly in the last decade. At the country level, decentralization and the subsequent separation of tasks between the management and operational levels seems to have spurred an increase in the number of guidelines. It is not clear whether this increase has been matched with reviews to assess the utility of these guidelines in influencing implementation.

Several reasons have been documented for the failure of guidelines to achieve their objectives, including inadequate consultation and consensus-building among stakeholders, lack of consideration of available resources, technical capacity, attitude and behavior of health professionals, tradition of using expert opinion-based approaches, lack of training on use of the guideline, lack of ownership, organizational barriers, and competing priorities [5,6,14-16]. Even when these broad strategic concerns are addressed, factors at the operational level such as lack of clarity, lack of familiarity with the content, and poor dissemination to end users derail the effectiveness of implementation [6,8].

The process of development, dissemination, implementation, and evaluation of guidelines has been shown to impact their effectiveness [4]. Schunemann *et al.* stated that a lack of standardized guideline development leads to widely varying recommendations [5]. Several organizations have provided guidelines for developing guidelines that spell out the components that must be incorporated [4,5]. Guidelines for the development of WHO guidelines consist of 19 components that deserve attention (Table 1) [3].

**Table 1:** Components highlighted in the protocol for development of WHO guidelines

**No.**	**Components**
1	Priority setting—addressing what guidelines need to be developed
2	Group composition and consultation – composition of the group that develops the guidelines and the consultation process
3	Declaration and avoidance of conflicts of interest
4	Group processes—how the group undertakes the process, leadership of the group
5	Identification of important outcomes
6	Explicit definition of the questions and eligibility criteria
7	Type of designs for different questions
8	Identification of evidence
9	Synthesis and presentation of evidence to inform guideline development
10	Specification and integration of values
11	Making judgments about desirable and undesirable effects
12	Taking account of equity
13	Grading evidence and recommendations
14	Taking account of costs
15	Applicability, transferability, and adaptation
16	Structure of reports
17	Methods for peer review
18	Planned methods of dissemination and implementation
19	Evaluation of guidelines

The framework in Table 1 was primarily developed for the WHO and works well for a global public health body with many more skills, resources, access to a large body of evidence, and partnerships than a low-income country such as Uganda. Low-income countries face specific situations that will make following these criteria a challenge, such as a lack of readily available evidence, limited capacity to synthesize and apply evidence, dependence on donors, limited domestic funding, an exaggerated role of civil society, and the chaotic nature of decision-making [17-21]. Systematic reviews, the recommended source of evidence, takes time, resources, and skills that may not be readily available in low-income countries [22-24]. Use of existing systematic reviews is an option, but still requires the establishment of structures to improve knowledge translation [20,25-27]. In addition, some components are more pronounced at a global level than at a country level, for example transferability of guidelines, variations in values, and legal standards. An analysis of WHO guidelines indicated that even within WHO, some of the reviewed guidelines fell short of these criteria [5,28-31].

Thomson *et al.*[3] proposed a framework highlighting the chain of events to produce effective guidelines: choice of topic; development group; development and presentation of guidelines; dissemination of guidelines; implementation of guidelines; and evaluation and revision of guidelines. Although this strategy is not as elaborate as the WHO framework, these steps are more focused on country-level processes and are more feasible in a low-income country like Uganda. In this study, we followed this framework as much as possible to assess how the process of developing guidelines in the Uganda health sector facilitates or abates their utility. Table 2 lists the facilitating factors at various stages of guideline development that must be taken into consideration. The literature, however, emphasizes that there is no international standard for guideline development, implying that there is room for country specificity [4,5].

**Table 2:** Factors that favor guidelines influencing practice

	
Development group	▪ Right people with appropriate skills in the group
	▪ Including representatives of people expected to implement the guidelines and beneficiaries
	▪ Working of the group – group process, dialogue, consensus building, effective leadership
Development and presentation of guidelines	▪ Are end users clearly defined?
	▪ Is the problem or issue clearly defined?
	▪ Is the presentation clear? Do we need to test for clarity?
	▪ How we should we present the guidelines for the different target audiences?
	▪ Will the chosen medium (booklet, posters, pocket card) be sufficiently durable?
Dissemination of guidelines	▪ Are we sure about the target users of the guidelines? How can we best reach them?
	▪ In what form should the guidelines be published and disseminated?
	▪ Systems of regular dissemination may be considered
	▪ Monitoring and evaluating dissemination
	▪ Planning for financial costs of dissemination
Implementing guidelines	▪ Having means to support implementation
	▪ Incentives to implement guidelines
Evaluation and revision of guidelines	▪ How will we know the guidelines have been received, read, respected, and locally promoted?
	▪ Methods required for assessment
	▪ Is there a clear means of evaluation?
	▪ Are there key indicators to measure implementation?
	▪ What is the expected outcome and how can it be measured
	▪ How often should the guidelines be reviewed or reformulated?
	▪ Who is responsible for initiating review?
	▪ How will reviewed guidelines be disseminated to replace redundant versions?

Adapted from Thomson et al. 1995 [3].

The main objective of this study was to describe guideline development, dissemination, monitoring, evaluation and revision within the health sector in Uganda, with a view to understanding how these processes facilitate or abate guideline utility. We assessed health planning, services management, and clinical guidelines using the framework in Table 2 as much as possible. We have drawn on the literature from a clinical perspective to assess the process of developing health services planning and management and clinical guidelines. Oxman *et al.* argued that clinical, public health, and health management guidelines require similar processes to ensure quality [32]. This study did not explicitly assess guideline implementation, but focused on the presence of factors that favor the ability of guidelines to influence health service delivery.

## Methods

### Study setting

**Figure 1:**
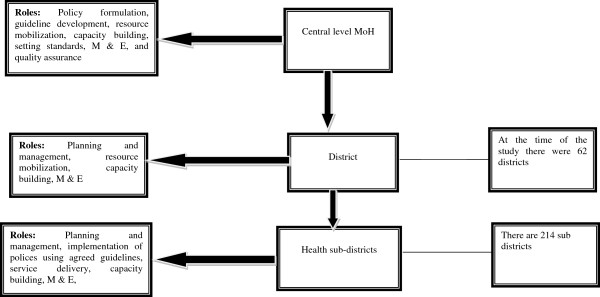
Managerial organization of health services.

The health system in Uganda is managerially organized at three levels (Figure 1); the roles and responsibilities of the different levels are well defined [33]. Policy formulation, guideline development, resource mobilization, capacity building, setting standards, monitoring and evaluation (M&E), and quality assurance are the mandate of the MoH (central level), while service delivery, planning and management, resource mobilization, implementation of polices using agreed guidelines, capacity building, and M&E are the responsibility of decentralized units (districts and health subdistricts in their areas of jurisdiction) [34]. The district, headed by a district health officer (DHO), is comprised of three health subdistricts on average, and the health subdistrict is headed by a health subdistrict in-charge.

**Figure 2:**
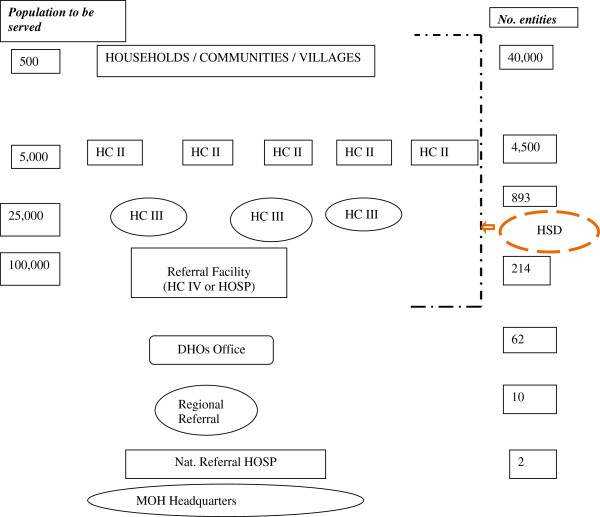
Organization of health service delivery.

Health services are delivered through a tiered system, as shown in Figure 2. The National Referral Hospitals provide complex specialist services and are involved in teaching and research. Regional Referral Hospitals provide referral services, specialized care, teaching, and research. The Health subdistrict is the health services delivery zone comprising a network of health centers (HCs) II and III and a referral facility (general hospital or HC IV). General hospitals provide general preventive and curative services. HC IV facilities provide curative and preventive services, emergency surgery, and blood transfusion services. HCs III and II, which are lower-level health facilities, provide mainly ambulatory services.

**Figure 3:**
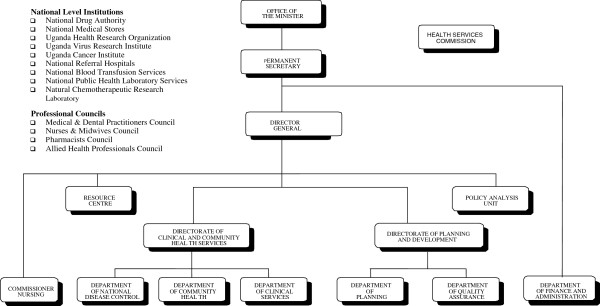
Organogram of Central level MoH.

Policy and guideline development is carried out by the MoH (central level; organogram in Figure 3). The Quality Assurance Department is responsible for coordination of guidelines development and maintains an updated inventory of all guidelines developed by the various departments. The actual development of guidelines is undertaken at the department level. The Policy Analysis Unit, on the other hand, is responsible for technical guidance on the relevance of guidelines and for ensuring that there is no contradiction with existing government policies.

National-level guidance on the development of polices and guidelines for the country is provided by the cabinet in the document ‘*Policy and Guideline Making in Uganda: A Guide to Policy Development*’ [35]. This guide details key principles of good policy-making and emphasizes early engagement of frontline workers involved in service delivery to help gauge what is deliverable and what matters to citizens. The policy and guideline development processes integrate the views of implementers through consultation and participation of service delivery-level workers right from the drafting process [35].

### Research methods

Qualitative and quantitative research methods were used for data collection and analysis. Qualitative aspects included review of documents, observation of guideline storage at all levels, and key informant interviews. Data were collected at the central (MoH), district, and service delivery levels. Quantitative aspects included counting of guidelines.

### Review of documents

All available guidelines were reviewed to ascertain: total number of guidelines; which departments had developed them; subject of the guideline; date of publication; group that developed the guideline; overlap in content and purpose; the impetus and process for guideline development; clear identification of end users; durability of the materials/format used to present the guidelines; and the process of consultation during guideline development.

### Selection of districts and health facilities

Table 3 contains details of selected district and health facilities. Twenty-two out of 62 districts were selected based on regional representation. Within the district, one health facility for each level of care was selected based on proximity to the district headquarters. Several of the selected districts did not have hospitals; in total, only five hospitals were sampled. Only 15 districts had HC IV facilities, 21 had HC III facilities, while HC II facilities were selected from only six districts.

**Table 3:** Selected districts and health facilities

**No.**	**District**	**DHO’s office**	**General hospital**	**HC IV**	**HC III**	**HC II**
1	Bullisa	1		1	1	1
2	Butaleja	1	1		1	
3	Bukedea	1		1	1	
4	Dokolo	1		1	1	
5	Gulu	1	1		1	
6	Isingiro	1		1	1	
7	Jinja	1				
8	Kabale	1		1	1	
9	Kabarole	1		1	1	1
10	Kampala	1		1	1	
11	Kamwenge	1			1	
12	Kapchorwa	1	1		1	
13	Kisoro	1		1	1	
14	Kumi	1	1		1	1
15	Lira	1		1	1	
16	Luweero	1		1	1	1
17	Masindi	1		1	1	1
18	Namutumba	1		1	1	
19	Nakasongola	1		1	1	1
20	Pallisa	1	1		1	
21	Sironko	1		1	1	
22	Oyam	1		1	1	
	**Total**	**22**	**5**	**15**	**21**	**6**

### Selection of key informants

Key informant interviews were undertaken at the central, district, and service delivery levels with respondents who were purposively selected. At the central level, managers of programs with the highest number of guidelines as identified at the document review stage were selected for interviews. Two officers were purposively selected from the Policy Analysis Unit and two from the Quality Assurance Department. At the district level, respondents were members of the District Health Team. The research team interviewed the DHO, who chairs the District Health Team, in addition to one randomly selected District Health Team member. At the hospital level, the research team interviewed the medical superintendent and the senior administrator. At HC II through IV facilities, the in-charge of the health facility, one randomly selected technical/clinical staff member, and one administrator were interviewed (except at HC II facilities, where administrative roles are undertaken by clinical staff). Some staff members were not available at the time of the survey; the final list of respondents included 102 technical officers and 14 administrators (Table 4).

**Table 4:** Details of the key informants selected for interview

**Central level**	**Respondents**	
	**Technical staff**	**Senior administrators**
**Central level**	**8**	-
Directors	1	-
Program managers	3	-
Officers from the Policy Analysis Unit	2	-
Officers from the Quality Assurance Department	2	-
**District level**	**28**	
DHO	9	
Other District Health Team members	19	
**Service delivery levels:**	**64**	
Hospitals (10)	6	4
HC IV (28)	22	6
HC III (34)	30	4
HC II (6)	6	-
**Total respondents**	**102**	**14**

Interviews were conducted using a semi-structured questionnaire, with each interview lasting one hour on average. At the central level, information was sought on the development group, development process, guideline presentation, dissemination, implementation, evaluation, and revision. Respondents were also asked to confirm the number of reviewed guidelines and comment on their content and relevance to the National Health Policy and Health Sector Strategic Plan. At the district and service delivery levels, our purpose was to ascertain the extent to which the key informants were consulted in the process of developing and reviewing guidelines, the availability of guidelines collated at the central level, and the key informants’ views on the utility and clarity of the guidelines.

Before the field visits, research teams comprising of social scientists, medical doctors, and public health specialists were familiarized with the purpose of the study and the questionnaire. The data collection tool was developed and pilot-tested in one district at the different levels of care (district, HC IV, and HCIII) and adjusted in accordance with the findings. Data collection took place between September and November 2008. Each team was led by a public health specialist who crosschecked the data at the end of each day. Gaps were filled in where they existed and clarity sought where required.

### Data analysis

Quantitative data were analyzed with Microsoft Excel, while qualitative data were analyzed using deductive content thematic analysis in line with a pre-conceived framework (Table 2).

### Ethical consideration

This study underwent a review by a team of officials from the MoH Quality Assurance Department, the Policy Analysis Unit, and the WHO country office (WHO) in Uganda. Furthermore, ethics review was sought from Uganda National Health Research Organization, which granted an IRB waiver for the following reasons: ‘the study being a routine assessment that will review the MoH policies and guidelines and is not testing or attaining a research hypothesis. The study aims to generate information for improving service delivery.’

## Results

### Overall study statistics

**Figure 4:**
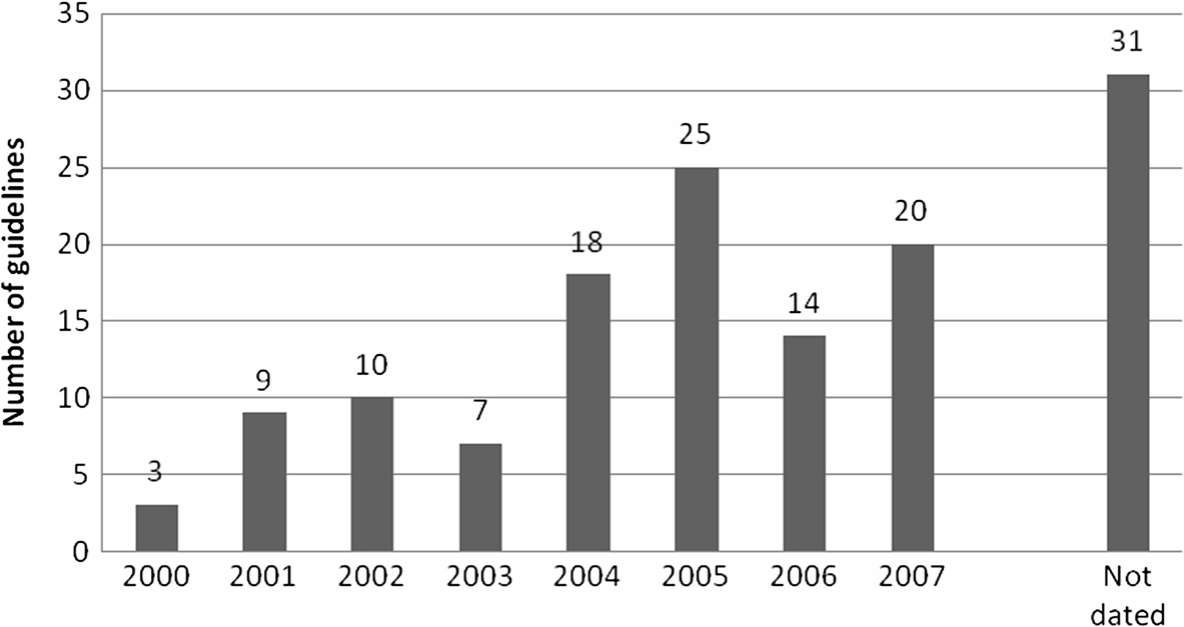
Number of guidelines by year of development.

There were 137 guidelines in the health sector by 2007 (Table 5). We noted an increase of slightly more than two-fold in the number of guidelines issued between 2003 and 2004 (Figure 4). Thirty-one (23%) of the reviewed guidelines were not dated, and it was not clear when they were developed. The three programs—Malaria, Human Immunodeficiency Virus (HIV), and Tuberculosis (TB)—under the National Disease Control Department accounted for 39% of the total number of guidelines. Programs under the Community Health Department accounted for 37% of the total number of guidelines. Of the individual programs, the HIV/AIDS, Malaria, Child Health, and Reproductive Health programs had the highest numbers of guidelines.

**Table 5:** Details of guidelines reviewed

**Guidelines by dept. & program**		**no. (%)**	**By year of development**	**no. (%)**
**Department**	**Community Health**		2000	3(2%)
**Programs** (37% of total)	Child health	18 (13%)	2001	9 (7%)
	Control of diarrheal diseases	3 (2%)	2002	10 (7%)
	Nutrition	11 (8%)	2003	7 (5%)
	Reproductive health	17 (12%)	2004	18 (13%)
	Zoonotic diseases	2 (1%)	2005	25 (18%)
**Department**	**National Disease Control**		2006	14 (10%)
**Programs** (39% of total)	HIV/AIDS	23 (17%)	2007	20 (15%)
	Malaria	18 (13%)	Not dated	31 (23%)
	TB/Leprosy	7 (5%)	**Total no.**	**137**
**Department**	**Quality Assurance**		**Guidelines by format**	**no. (%)**
**Program** (5% of total)	Quality assurance	7 (5%)	Booklet	109 (80%)
**Department**	**Clinical Services**		Chart	18 (13%)
**Programs** (10% of total)	Mental health, disability prevention, and rehabilitation	13 (9%)	Leaflet	8 (6%)
	Clinical division	1 (1%)	Desk aides	2 (1%)
**Department**	**Finance and Administration**		**Total no.**	**137**
**Program** (4% of total)	Finance and administration	5 (4%)		
**Department**	**Planning**			
**Programs** (9% of total)	Human resources	2 (1%)		
	Planning department	1 (1%)		
	Resource center and surveillance division	9 (7%)		
	**Total no.**	**137**		

### Development group

Guidelines were developed by officers working on different programs and/or in different departments. These senior health professionals possess relevant qualifications and advanced degrees, for example in public health, social sciences, environmental health, economics, and specialized medical disciplines.

All respondents at the central level reported that efforts were made to consult all stakeholders, including implementers, in the development process, although document review revealed a varying degree of consultation. Consultations also took place with related line ministries, including the Ministries of Education, Public Service, Finance and Economic Development, Labor, Gender, and Social Development, and Environment and Water. There appeared to be a clear consultation process; the development process included reference to existing national strategic documents such as the Constitution and the National Health Policy, and a range of relevant stakeholders within and outside the sector were engaged. For 40% of the guidelines, developers had consulted stakeholders at the district and service delivery levels. In a majority of cases, consultations were limited to national-level stakeholders. Representatives of civil society organizations had been consulted in almost 42% of the guidelines, and the understanding was that these personnel represented communities/beneficiaries. However, the representatives of civil society were based at the national level, and it was not clear to what extent they gathered views from the communities. Respondents at the district (57%) and health facility levels (61%) expressed a lack of user involvement in the development process, highlighting the absence of a bottom-up approach as contributing to the development of ineffective guidelines. Other stakeholders, especially at the service delivery level, were often excluded, even though they are considered to be vital for the development of realistic and practical guidelines. The consultative process was also reported to exclude existing administrative structures such as the local governments in which district health services are embedded, making it difficult to implement guidelines and to attract the support of local government officials when requested. One DHO remarked that:

‘Guidelines fail to influence the agenda at the service delivery level [and yet] everyone should know the content and implication of health guidelines. There is little input from sub-national levels, leading to unrealistic guidelines.’

### Development and presentation of guidelines

Of the reviewed guidelines, 78% articulated the aspects of service delivery that were to be strengthened, as well as the impetus for developing the guidelines. Target users were generally defined as health workers, without categorization by cadres or level of care. Only 38% of reviewed guidelines clearly defined target users.

### Conflict

Several of the guidelines duplicated content, and some conflicted with each other. Overlap in content was reported for certain guidelines, including Reproductive Health, HIV/AIDS, Anti-Retroviral Therapy, and Prevention of Mother-to-Child Transmission (PMTCT). This finding was corroborated by document review, which revealed considerable overlap in the content and purpose of guidelines within and across departments. Examples of such documents included: *Infection Control and Prevention Guidelines (2004, 2nd ed.)*, produced by the AIDS Control Program, *Policies and Guidelines on Infection Control (2005)*, produced by the Quality Assurance Department, *TB Infection Control Guidelines (2007)*, produced by the National TB/Leprosy Program, and *Infection Control at Health Facilities—Management of Epidemic Diarrheal Disease Outbreaks (2005)*, produced by the Community Health Department. The overlap of purpose and content also occurred within departments. For example, the Community Health Department published, *Oral Health Guidelines for Teachers in nursery and primary schools, (School Health Series, 1st Ed, 2002)*, as well as *Oral Health Guidelines for schools, (not dated)*. The Nutrition Division released *National Guidelines on the Management of Severe Malnutrition, (not dated)*, *National Guidelines on the Management of Moderate Malnutrition at supplementary feeding centers (August 2005)*, and *Management of Severe Malnutrition in Uganda: Guidelines on Specific Needs of Therapeutic Feeding Centers (2005)*.

The majority of guidelines were presented as booklets (80%), with a few presented as charts (13%), leaflets (6%), or desk aides (1%). Respondents at the central level reported that desk aides and charts were meant for quick reference, and should be given to all health workers irrespective of the level of care and training. These respondents also reported that there was no systematic mechanism for pre-testing guidelines for clarity before finalization. One district member mentioned the internet as an alternative source of health service guidelines, which were accessed through the MoH website. While some of the guidelines were published electronically and in paper format, the latter was more common. In all cases, dissemination emphasized the paper format.

At the service delivery level, respondents reported that charts were more convenient to use because of the simple language and illustrative diagrams. For this reason, charts for the integrated management of childhood illnesses, malaria treatment, and syndromic management of sexually transmitted infections were considered to be the more appropriate format for lower-level units such as HC II facilities, as elaborated in the following quotes:

‘A chart in front of a health worker is a ready reference; otherwise one can easily miss signs and symptoms.’ Clinical officer at HC IV.

‘A chart is sometimes better than someone explaining, and they are also good for health education.’ Health worker at HC II.

Although charts were the preferred format, they were not properly mounted on the walls for display in nearly all health units visited, and therefore the charts exhibited a tendency to fall and to damage the underlying wall.

Several recommendations for improving guideline clarity were provided by district-level and service delivery-level respondents. Twenty-nine percent and 82% of district-level and lower-level respondents, respectively, mentioned that guidelines should be translated into local languages. Fourteen percent of district-level respondents reported that every guideline should have a training manual to explain its use, thus improving clarity. There were conflicting views on the level of detail expected in the guidelines. Some respondents expected guidelines to be brief and user-friendly. Others preferred detailed guidelines that covered common conditions as well as emergencies.

### Dissemination within the MoH and to the districts

The Quality Assurance Department, which should have a copy of all developed guidelines, had less than 60% of the documents available at the departmental level. A number of these documents were shelved in stores and offices, lying unused. Departments lacked storage facilities for easy document retrieval, and guidelines were piled in the central store, calling into question their utility or need for development in the first place.

Forty-eight percent of respondents at the central level reported that there was no clear dissemination process for newly developed guidelines. Lack of funds for dissemination was cited as the main hindrance at both the central and district levels. Fifty-two percent of respondents at the district level noted that the dissemination process was unclear and was occasionally untimely; in some cases, documents were received when they were already out of date. It was felt that dissemination of documents should receive the same level of priority as other medical supplies. One DHO remarked that:

‘The problem is in dissemination—they remain at MoH headquarters. Only a few are disseminated. The only chance for dissemination is when an issue is on the agenda; up to 90% of documents are not [available] at [the] district level. If they are not at the district level, how can health subdistricts be expected to access them, let alone implement them?’

Two main modes of dissemination to the district level were noted. One mode involved giving out copies of the guidelines to district officials attending MoH workshops, while the other method was to courier copies directly to the districts. Occasionally, copies of the guidelines were handed to district personnel when they visited the MoH headquarters for meetings or other assignments. Respondents found the workshop dissemination mode to have some shortcomings. One DHO remarked that:

‘A distribution system [for service guidelines] is lacking—if there is no workshop, districts may not get copies; there is [a] need for a clear system for dissemination and storage right from the center to districts, e.g., there is [a] need for an inventory to determine which guidelines have been distributed to which district.’ At the service delivery level, a health worker at HC IV stated that ‘at this facility, these guidelines are not collected from the MoH […] I have my own copy but I teased them [the managers] and requested a copy of the Uganda Clinical Guidelines for the unit in 2006, but have since not obtained a copy.’

Within the district, the respondents identified three main concerns regarding the process of guideline dissemination to lower levels. First, there were rarely enough copies for all lower-level facilities. Second, documents handed to some health subdistrict in-charges tended to remain at that level, and were not disseminated to lower-level facilities. This issue was thought to be related to the management performance of the health subdistrict in-charge, as illustrated by the following quotes:

‘Often the health subdistrict in-charges do not implement what is agreed on, e.g., do not report back on work-plan implementation, and yet they get the money. They are never available at workstations, yet they control resources. Immunization outreaches are not done; health workers are not paid for outreaches. Could this be the result of weak management skills? The health subdistrict policy needs to be reviewed—the assumption that all doctors are good managers is not true. Senior clinical officers have proven better in some cases. A medical officer can control resources and frustrate everyone.’ DHO.

‘Guidelines in the lower-level units never reach the health workers because the in-charges individualize them.’ District Health Team member.

‘I have never seen any guidelines. The former in-charge personalized them and used to keep them at home. One time I wanted to prepare a talk on health unit management committees, but could not even trace a copy.’ Health worker, HC IV.

The third concern was the inability of some districts to effectively disseminate guidelines. Sixty-two percent of respondents at the district level noted the need to support guideline dissemination by districts, including provision of adequate copies, training of health workers on how to use the guidelines, and follow-up supervision by the MoH after guidelines were disseminated. One DHO mentioned that:

‘When guidelines are formulated and handed out, there is no orientation of staff and inadequate copies are given out, e.g., PMTCT: new drugs were introduced to the treatment regimen and yet staff were not oriented. Other examples are in ART [anti-retroviral therapy] [and] child counseling. It is difficult in such circumstances to implement changes when no orientation has taken place and only one guideline has been given to the whole district.’

At the service delivery level, 78% of respondents felt that there were inadequate copies of the health service management guidelines. The district with the most documents had less than 40% of the 137 guidelines at the MoH, a number that was less than 20% at the health subdistrict level. On the other hand, respondents perceived that there were too many documents relating to certain areas, such as PMTCT. Respondents further stated that at the district and health subdistrict levels, there should be a complete set of guidelines for each area of service delivery, regardless of the frequency of reference to a document. One health worker in a health subdistrict stated that ‘even if a document is just for reference and [is] not in frequent use, there is still a need to have all.’

There are examples of good practices to improve awareness and access to health service guidelines. In one district, all new guidelines were brought to the attention of the District Health Team at the monthly staff meeting. Some of the health facilities reported organizing continuing medical education sessions to keep staff updated, but it was felt that higher profile sessions organized by the district would be more motivating. One HC III health worker stated that:

‘If the district were to organize workshops for lower staff cadres this could motivate them [to read the guidelines]. Continuous medical education sessions organized by the DHO outside the health facility should include nurses, as there is no feedback from the other staff who attend these sessions.’

There was no systematic process for monitoring and evaluating the dissemination of guidelines at both the national and district levels.

### Implementing guidelines

Not all guidelines were being implemented, for a number of reasons. Among the constraints was limited funding to ensure the availability of the required inputs. One DHO remarked that ‘the new malaria treatment policy cannot be implemented adequately—Coartem© as a first-line anti-malarial is not available in adequate [amounts] to support compliance.’ Forty-two percent of respondents at the district level noted the disconnect between the MoH and the decentralized levels. One DHO stated that:

‘Decentralization detached lower local governments from the center. The MoH sees their role as developing policies and guidelines, but who are they making them for? No one sees to it that there are enough resources to implement these policies and guidelines. When [the] MoH makes guidelines and they look for resources, this money remains at the central level’

The mismatch between policy expectations and the reality on the ground was also mentioned. One DHO remarked that:

‘The human resource policy is not cognizant of the staffing needs, e.g., recommended staffing norms of four midwives for a HC IV with a theater and maternity ward are inadequate. Our HC IV conducts 160 normal deliveries per month and attends to about 500 ANC [antenatal care] new clients and re-attendances. We have adjusted this to 10 [midwives], but the staff are still overstretched.’

Failure of guidelines to account for potential conflict with other related sectors was also cited as a hindrance. Guidelines from other sectors can be in conflict with those of the health sector, sometimes putting health sector managers employed by local governments in a dilemma as to which guidelines to follow. At times, solutions adopted by health sector managers to solve this dilemma conflict with other regulations. The following quotation from a DHO highlights this challenge:

‘The fiscal decentralization strategy led to collapsing of bank accounts at the Local Government level in order to minimize expenditure in bank charges. This meant that health units (institutions by right of having an in-charge, work plan, and budget) had to close accounts and transactions conducted from a central account. The result is that now health unit money is given to individuals by checks in their names. There is [a] loss of money during the banking process, which is not accounted for, and the temptation to divert funds for personal use is strong. Moreover, keeping public funds on a personal account is against the financial accounting regulations.’

Some DHOs stated that the absence of a reading culture constrains the utility of health service guidelines. In one hospital, guidelines in the office of the medical superintendent were covered with dust, and the officer was not sure when they had been received. Health workers at the same hospital were aware that guidelines were available, but confessed that they had not read them.

It was also felt that the utility of service guidelines could be improved if staff handling the day-to-day services under the relevant programs had prior update training on the use of the guideline. A HC IV midwife stated that:

‘Voluntary Counseling and Testing is offered as an outreach to this health facility by the DHO staff, and yet none of us who deal with the patients on a day-to-day basis have been trained. We would also like to be updated on this service.’

### Evaluation, revision, and review of guidelines

The majority of respondents at the MoH level stated that there was no mechanism for ensuring that guidelines are received, used, and promoted at the service-delivery level. The means of evaluation were not stated, and there were no indicators to measure guideline implementation. Criteria for reviewing guidelines were not in place, and the person to initiate the review was vaguely referred to as ‘the department responsible.’

The mechanism for disseminating revised guidelines to replace old ones was not established. There were several outdated and draft documents in circulation. Districts had various versions of the same guideline with different production dates, for example guidelines addressing PMCTC for HIV that were published in 2001, 2003, and 2006. There were no references to earlier versions, and it was not clear whether more recent guidelines were addenda or were intended to replace the old guidelines. Some documents (23%) lacked publication dates, and it was difficult to know whether a guideline was current or outdated. One DHO stated that ‘some of the guidelines are in draft form, and one is not sure whether to consider it as a pre-test or [a] final version.’ The process for testing, withdrawing, and introducing guidelines was not laid down explicitly.

The system for reviewing guidelines with reference to the responsible center was in place. Respondents were aware that the Quality Assurance Department was responsible for coordinating this process. However, departments were not following this protocol for several reasons including, time constraints and the weak performance of the Quality Assurance Department. The Quality Assurance Department is understaffed, which compromises their ability to execute their mandate. The need for regular guideline updating was noted by 72% of respondents at the MoH level, for reasons such as the emergence of ‘new’ diseases such as Ebola virus disease, rapidly changing medical technology, and new medicines.

## Discussion

This study has shown that there are numerous health service guidelines in the health sector in Uganda, several of which overlap in content and purpose. We noted that programs targeted by health-related Millennium Development Goals (malaria, HIV, reproductive health, and child programs) had the highest number of guidelines. The special attention paid to these programs to achieve the Millennium Development Goals may have sparked guideline development in an effort to improve the delivery of health interventions. We also identified a substantial increase in the number of guidelines beginning in 2003, when Uganda started benefiting from the Global Fund Against HIV, TB, and Malaria [2]. In the same year, Uganda also began to receive funding from the President’s Emergency Fund for AIDS Relief, and USAID/President’s Malaria Initiative funding was directed to Uganda starting in 2006 [36]. Increased funding enabled the scaling up of health interventions, and guideline development may have been seen as an input to scaling-up service coverage. On the other hand, these grants were time-bound, and guideline development may have been an activity that could be implemented rapidly, demonstrating the country’s absorption capacity. This high number of guidelines hinders their use; as noted by Armstrong that large numbers of guidelines may be overwhelming to any user, impacting negatively on their use [14]. In addition, guideline development consumes both time and resources, requiring the sector to lay down explicit criteria to decide which areas need guidelines [29].

In terms of the nature of the teams within guideline development groups, we found that they were multidisciplinary and that, furthermore, they engaged with key stakeholders from other disciplines. Consultations also took place with related line ministries. Involving all important stakeholders and beneficiaries as much as possible, especially individuals with the right skills, enhances guideline acceptability, ownership, and credibility [8,31,37]. Multidisciplinary teams, help to balance individual biases resulting in more valid guidelines [31]. Although the team members at the MoH were skilled in their respective areas, we did not ascertain whether the stakeholders being consulted had the necessary skills to support the process. Training sessions are necessary for the people responsible for guideline development, especially in low-income countries harboring stakeholders of varied capacities. Effective leadership of the group has been noted to be crucial [31]. In our study, the team leaders were senior MoH officials heading divisions or departments. We however did not assess their leadership of the guideline development process. The leader must be neutral and have the capacity to facilitate team spirit, consensus building, collaboration, and engagement of relevant stakeholders [30,31].

This study has also revealed a limited involvement of operational-level users of health service guidelines. Reasons for lack of adequate consultation during the development process could be several; if funding from Global Health Initiatives is used to develop guidelines, the time bound nature of these grants may not allow enough time for consultation. On the other hand, inadequate consultation may be the result of the lack of an established, systematic process for consultation. Excluding a wider range of stakeholders risks limiting guidelines to technical concerns without addressing the broader environment, as evidenced by key informant responses about the ineffectiveness and/or impracticability of some of the guidelines. Insufficient active user and relevant administrative structures involvement, are among the documented barriers to guideline use [38]. It is important to consider gaining the participation of those with the power and authority to implement guidelines or to persuade others to do so [4]. Some researchers have urged for the consideration of consumer/community values during guideline development [37]. Challenges of achieving effective community involvement have been identified, and focus group discussion has been highlighted as an approach to integrate the community into guideline development [37]. However, this approach may be costly in resource-constrained settings. In our study, we found that community integration is implied in civil society involvement; strengthening the capacity of civil society to gather the views of the community may be a cheaper option.

Regarding guideline development and presentation, we noted a poor definition of end users, with a tendency to view users as one group irrespective of their training. Guidelines need to be tailored to the cadre and technical plateau of the different levels in the healthcare delivery system. Users operating at a lower level of care appreciate simplified language and a visual presentation that provides a quick reference, as opposed to booklets. Some organizations use different formats for different types of guidelines, others produce various versions of the same guideline, and others have a standard format for all guidelines [32]. A case has been made for target audience-tailored formats; we also urge that the format be tailored to the level of care, taking into consideration the technical capacity at the different levels of the healthcare delivery system. Health workers identified the need for training manuals on how to use guideline, which may point to complicated guideline presentation and content, lack of clarity, or both. Insufficient clarity and capacity for implementation, especially at lower levels, were cited as factors affecting the use of guidelines. Schunemann *et al.* also discussed the need for detailed manuals to enhance guideline use [5]. However, even a detailed manual may not suffice, because it is impossible for a nationally developed guideline to cover all operational details for all implementation settings. In addition, other researchers caution that guidelines need to be clear and easy to understand, without much reference to other supporting materials [32,39]. Francke *et al.* found that guidelines that were easy to understand had a large chance of being implemented [15]. Some studies have raised the issue of guideline-related barriers affecting use, specifically complexity and whether a behavior is being eliminated or added [40,41].

This study has revealed poor dissemination and unavailability of guidelines where they should be implemented. Many of the guidelines were in storage at the central level, calling into question the need for their development in the first place. Evidence shows that access plays a role in improving use [5,6,42]. We found that dissemination strategies were largely passive and unclear, and guidelines were frequently distributed at workshops that did not necessarily address the issues outlined in the guideline in question. Studies have shown that passive attempts to distribute information have little success [6,38,39,42,43]. This issue is compounded by the lack of a reading culture, which further negatively impacts on guideline use even where they are available [20]. Organizing training sessions on new guidelines has been shown to enhance guideline uptake and implementation, and some organizations have used education materials and workshops as part of their guideline implementation strategies [6,39,42,43]. Some researchers however caution that groups must be small, focused on the topic, and multiple training methodologies used [39]. This strategy can be further mainstreamed in supervision where supervisors explain guidelines and can possibly take the initiative to organize local seminars for HC and hospital staff at the district or even subdistrict levels. Insufficient awareness and a lack of familiarity with existing guidelines and their content have been documented as barriers to guideline use [15,44]. Multifaceted interventions targeting different barriers to change are more effective than a single intervention; a combined strategy of training, supervision, joint consultation sessions, audit, and passive dissemination would be more effective than any element implemented in isolation [16,39,45,46]. However, this integrated strategy has cost implications that may challenge low-income countries.

Under implementation, this study also found that reference to guidelines was varied. Some existing guidelines did not account for all possible scenarios, likely due to the exclusion of key stakeholders during guideline development. Consultation with all relevant stakeholders, including users of guidelines, is crucial for effective guideline utilization [29]. Consultation enhances participation of stakeholders and subsequently promotes ownership [7]. Other factors known to facilitate guideline uptake include provision of incentives to implementers and health-worker attitude toward the guideline, both of which can also be enhanced through consultation [16,29,39,42,47]. The poor consultation process possibly contributes to the conflict with other government sector/local government guidelines. In decentralized settings where power lies with local governments, efforts must be made to align sector guidelines to local government guidelines. Furthermore, resources and the broader environment within which guidelines are expected to be implemented need to be taken into consideration. Resnicow *et al.* raised the issue of environment-related barriers to guideline use; they noted that guideline implementation may be affected by factors not under the implementer’s control like availability of resource, required inputs such as medicines and staff, which we also found in our study [48]. Feasibility of implementation, required organizational changes, affordability, and acceptability of the guidelines must be considered [7,8,29,49]. In a systematic review of integrating primary healthcare in low- and middle-income countries, authors noted that in success cases, required inputs were provided alongside guidelines which enhanced their implementation [50]. Another systematic review of improving outpatient referral from primary to secondary care also noted that the referral process improved if guidelines for referral were provided along with referral forms [45].

Under evaluation of guidelines, respondents at the service delivery level pointed to the need for continuing support supervision after guidelines were distributed, stating that guidelines should not replace the needed supervision. The MoH established area teams, which comprise of multidisciplinary teams overseeing a group of districts in a consistent manner. These would ideally supervise and evaluate implementation of guidelines as well. Performance of these teams has been suboptimal for several reasons including lack of funding, logistical challenges, lack of effective follow-up on issues, and inadequate human resources [51]. The challenges of undertaking effective supervision have been raised in the literature, and include a lack of tools, logistics, and support from superiors, as well as being burdened with administrative responsibilities [39]. The need for supervision and audit to enhance guideline implementation has been documented in several studies [6,39,52]. Supervision would provide feedback on clarity and utility of guidelines as a way of guiding improvements in format and consultation. Some researchers have highlighted the multiple benefits of supervision in improving guideline uptake, such as professional development, improving job satisfaction, and enhancing motivation [39]. At the utilization level, there was no consensus on the level of detail expected in the guidelines, which may be partly explained by the different levels of training of the respondents. There is currently no clear means of evaluating whether guidelines are being implemented and no means to measure outcome. Schunemann *et al.* underscored the challenge of building consensus on which outcomes are most important [53]. Thomson *et al.* also caution about measuring outcomes, noting that it is impractical and complex to do so. They suggest that outcome measurement should instead consider the entire process of development, dissemination, implementation, evaluation, and review, because failure can occur at any of these steps [4].

Under review of guidelines; although the Quality Assurance Department is responsible for coordinating the development and review of guidelines, this study has shown that this process is not centralized, with departments and programs developing guidelines without involving the coordinating unit. The overlap in content and purpose seems to be linked to the decentralized nature of the development process, which occurs without a central monitoring/checking mechanism. Although a majority of respondents identified factors that would necessitate revision of guidelines, the criteria were not explicitly laid out. Shekelle *et al.* stated that guidelines should include a scheduled review date, although they again caution that this may lead to premature guideline revision, especially if changes in a given area are not rapid and/or use of outdated guidelines in fast-changing fields [54]. It may be reasonable to reassess validity every three years after publication, with room to incorporate smaller, earlier updates if required [5]. Additional considerations may include the emergence of new diseases and availability of new evidence [29].

### Limitations of the study

There are important parameters that were not assessed in this study, including the use of evidence in guideline development and the management of conflict of interest in the development group. Evidence has shown that locally developed guidelines are more likely to be implemented compared to guidelines developed in response to intervention by international organizations, but we did not investigate this aspect. However, we believe that we have identified important issues that can guide future improvements in guideline development and subsequent utilization in a low-income country.

## Conclusion

The development of guidelines consumes resources, and to ensure a return on investment the guidelines must achieve their intended objective. Low-income countries need to appreciate that the process of guideline development and review is both time- and resource-consuming. Achieving guideline effectiveness is partly predicated on the process followed in development. This study has shown that the process of developing, disseminating, and implementing guidelines needs to be improved to enhance their utility. There are some aspects that can easily be handled at a country level, while a regional approach may be beneficial for other aspects, given the potential to pool expertise and financial resources.

At the country level, there is a need to develop and adopt a standard guide for developing guidelines in the health sector. The process must be consultative; guidelines must be disseminated, enforced, and accompanied by the required capacity building and inputs for implementation. Adaptation of the WHO handbook for guideline development to specific country contexts may be considered. Committed teams with necessary skills and leadership, a system for dissemination, routine monitoring of use of guidelines, introduction of new guidelines, withdraw of old ones and criteria for revision must be in place. The option of strengthening civil society to harness the contributions of communities and beneficiaries in guideline development should be explored. Guideline development and implementation must be planned for and resources mobilized. Required funding must be mobilized within government budgets and/or project proposals developed to access health grants if a guideline is expected to be developed.

At the regional level, regional professional bodies, the WHO inter-country support teams and the Africa Regional Office can support the development of high-quality, evidence-based guidelines for countries in the region by fostering evidence generation, synthesis, and capacity building. WHO may also create a repository of guidelines to serve as a resource if a country needs to develop a guideline similar to an existing guideline from another country in the region. The regional level can also provide technical guidance in areas, such as external validation mechanisms and the development of possible methodologies for assessing guideline use that countries in the region can adapt.

## Abbreviations

DHO: District health officer; HC: Health center; M & E: Monitoring and evaluation; MoH: Ministry of health; PMTCT: Prevention of mother-to-child transmission; TB: Tuberculosis; WHO: World health organization.

## Competing interests

The authors declare no competing interests.

## Authors’ contributions

JNO contributed to the conception and design of the study, data analysis and interpretation, and led the drafting of the manuscript. JBW contributed to the conception and design of the study, data interpretation, and participated in the drafting of the manuscript. SKB contributed to the conception and design of the study, data analysis and interpretation, and participated in the drafting of the manuscript. BC contributed data analysis and interpretation and participated in the drafting of the manuscript. All authors reviewed and approved the final manuscript.
